# Awareness and integrated information theory identify plant meristems as sites of conscious activity

**DOI:** 10.1007/s00709-021-01633-1

**Published:** 2021-03-21

**Authors:** Anthony Trewavas

**Affiliations:** Institute of Molecular Plant Science, University of Edinburgh, EH9 3JH Edinburgh, Scotland

**Keywords:** Plant consciousness, Awareness, Consciousness, Integrated information theory, Meristems, Associative learning

## Abstract

Lacking an anatomical brain/nervous system, it is assumed plants are not conscious. The biological function of consciousness is an input to behaviour; it is adaptive (subject to selection) and based on information. Complex language makes human consciousness unique. Consciousness is equated to awareness. All organisms are aware of their surroundings, modifying their behaviour to improve survival. Awareness requires assessment too. The mechanisms of animal assessment are neural while molecular and electrical in plants. Awareness of plants being also consciousness may resolve controversy. The integrated information theory (IIT), a leading theory of consciousness, is also blind to brains, nerves and synapses. The integrated information theory indicates plant awareness involves information of two kinds: (1) communicative, extrinsic information as a result of the perception of environmental changes and (2) integrated intrinsic information located in the shoot and root meristems and possibly cambium. The combination of information constructs an information nexus in the meristems leading to assessment and behaviour. The interpretation of integrated information in meristems probably involves the complex networks built around [Ca^2+^]i that also enable plant learning, memory and intelligent activities. A mature plant contains a large number of conjoined, conscious or aware, meristems possibly unique in the living kingdom.

## Introduction

It has been claimed that the lack of a defined brain and nervous system in plants indicates they cannot be conscious organisms (Mallatt et al. 2021; Taiz et al. 2019). While an obvious anatomical plant brain can be dismissed, its functional equivalent, of whatever small or large capability, cannot. Sir J.G. Bose FRS in his book, The Nervous Mechanism of Plants (1926), experimentally demonstrated a defined plant nervous system. He identified the phloem as conducting action potentials, established its latent period as about 0.06 s and moving with a velocity of some 12–40 cm/s. He reported experimental evidence for synaptic behaviour that enabled electrical movement in only one direction, demonstrated conductive fatigue on constant repetition and observed facilitation of transmission; a priming ineffective stimulus now becomes effective. He concluded that the characteristics of nerve conduction were similar to those in animals and that the phloem was the plant nerve.

This article instead is centred about a more difficult question for plant biologists: that of consciousness. Human consciousness dominates most discussion and research (Griffin 1976; Koch 2019). Visual (and mental) images, thoughts, feelings and emotions are familiar, and these are easily recognised because words are available to describe them. Mankind is the only known species that can do that. Human linguistic ability is unparalleled in the biological world. The Oxford English dictionary, for example, contains half a million words. Human consciousness is probably learnt from the foetus onwards, into birth and through childhood. Edelman’s neural Darwinism (Edelman 1993, 2003) and his theory of neuronal group selection represent creative approaches to understanding this complex process.

Is human consciousness unique? Many feel that language is the game changer. ‘The distinction between men and animals is in one sense only a difference in degree. But the extent of the degree makes all the difference. The rubicon has been crossed’ (Whitehead 1938). Waddington (1972, p. 143) in a discussion on consciousness and mind posed a fundamental problem. ‘If consciousness were to be adopted as a criterion of mind it would be a signally useless one, because the only way to tell whether any other thing is conscious, is to ask it. And that you can only do to human beings. The concept of consciousness is not applicable to anything but a language- using animal’. ‘There is a complete conceptual gap between any type of material interaction and self-awareness and one conclusion that has been drawn for instance by Whitehead, was that as we know there is consciousness somewhere, namely in ourselves, then something in some way akin to it, must be present in all entities; not that all entities have a consciousness as developed and evolved as our own. That is a logical argument that I find very difficult to refute’. Plants represent more than 99% of life on earth (Trewavas 2014). *All entities* includes them too.

If the nature and complexity of human consciousness are indeed unique, then it will not exist in any other animal species let alone plants. But some form or kind of consciousness is not dismissed by Waddington (1972); it will be much simpler and less developed than that of mankind but does need definition.

## Replacing ‘consciousness’ by ‘awareness’ clarifies understanding

In the controversy over plant consciousness, it is essential to state what the authors mean by consciousness, and such descriptions seem lacking in both Taiz et al. (2019) and Mallatt et al. (2021) particularly when discussing animals. The assumption in this article is that human consciousness is unique because of complex language which animals do not possess. What out of mental thoughts/images or visual images, feelings, emotions and sentience do the authors think such animals have and what evidence is offered that convincingly demonstrates their presence? This section seeks to identify awareness as an operational version of consciousness based on behaviour and thus is potentially measurable.

There are two problems with the word consciousness. First is the issue raised by Waddington (1972). If you can’t ask it, any assumptions of consciousness are speculation. Mallatt et al. (2021) claim large numbers of animals are conscious, none of which can provide a verbal answer. Second, when the issue of plant consciousness is raised, most plant biologists simply assume it is the human version of consciousness that is being referenced and rightly reject that possibility. If human consciousness is unique, then why not limit the use of the word ‘consciousness’ only to its well-known human version? That is the view taken here.

The alternative for every other organism bar mankind could be ‘awareness’. Stuart Sutherland (1996) in his International Dictionary of Psychology states that ‘to be conscious it is only necessary to be aware of the outside world’. The consciousness expert Koch (2002, p. 2; 2019, p. 1) considers that consciousness and awareness are interchangeable with a preference for awareness. Griffin (1976), in his The Question of Animal Awareness, uses consciousness and awareness, interchangeably stating that awareness influences behaviour and is adaptive. The plant biologist, Carl Leopold (2014), equated awareness directly with plant consciousness and quoted behaviour too. ‘In the simplest sense, consciousness is an awareness of the outside world’ (Margulis and Sagan 1995). See also numerous statements and references on awareness as consciousness in Thompson (2007) and Blackmore (2017).

All organisms in natural circumstances, animals, plants and bacteria, are aware of changes in their environment and respond by changing behaviour. Those changes in behaviour hinge around adaptive change and natural selection; the aim is to improve survival. Earl (2014) states that consciousness, ‘can only have biological value as an input to a mechanism or mechanisms that determine behaviour’, is adaptive, subject to selection, improving the probability of individual survival in the challenges of the real world.

For awareness to influence behaviour, an assessment (appraisal) of the detected external, or internal, signal is essential to determine how and where to respond. Whereas higher animals mainly use nervous mechanisms, brains and mental images for assessment, higher plants use molecular mechanisms: hormones, genomic changes and a complex system revolving around electrical changes and [Ca^2+^]_i_. Learning, memory and intelligence also contribute to the process of assessment because they influence behaviour. Their contribution to assessment in plants has already been reported (Calvo et al. 2020a, b; Calvo and Trewavas 2020).

It is feasible to place all organisms on a scale of awareness based on observations of the degree of complexity of behaviour. For example, Ginsburg and Jablonka (2010) state that during evolution, behaviour becomes more complex, and they quote the evolution of open-ended associative learning as an example (Bronfman et al. 2016). Some protozoa exhibit conditioned behaviour, but the numbers of such behaviours may be limited (De la Fuente et al. 2019).

Associated learning is common in higher plants. There are several forms of associative learning (Abrams and Kandel 1988). Classical conditioning is particularly important, because it represents the simplest case of learning the association between two events (Abrams and Kandel 1988). Associative learning and memory can drastically speed up rates of adaptation by triggering the organism to use the solution of one problem to solve a new but similar problem. However, coupling two events together can improve survival by predicting the likely onset of a stressful condition in which prior preparation improves survival.

Abrams and Kandel (1988) describe two essential criteria for classical conditioning. Firstly is *temporal contiguity*, in which one conditioning event precedes the other conditioned event. Secondly is *contingency***,** in which the predictive relationship is learned because the two events are positively correlated. I have indicated a few here; there are undoubtedly more. Those that can be identified as associative are ABA and light (Goh et al. 2003), growth and temperature (Gagliano et al. (2016), between red and blue light impacts on phototropism (Curry 1969), drought and cold tolerance (Medeiros and Pockman 2011), photoperiodism and leaf abscission (Olmsted 1956) and mechanical stimulation and frost and drought resistance (Suge 1980; Jaffe and Biro 1979). The first signal acts as the conditioning stimulus, and the second acts as the conditioned event to be joined to the first stimulus. However, these associatively learnt behaviours will depend on the ecological origin of the species, will vary in their skill of application with the individual and depend on the stage of development and specific environmental circumstances in which it occurs. Associative learning enables plants to recognise adaptive and predictive relationships within their environment.

Abrams and Kandel also identified [Ca^2+^]_I_ as the convergent signal between the two events that cause the linkage they examined, and this seems likely to be the case in the plant examples above.

The advantages of replacing consciousness by awareness:

1. The sterile argument claiming plants are not conscious is avoided (e.g. Taiz et al. 2019; Mallatt et al. 2021). Both animals and plants are aware, and given the relation between awareness and consciousness, plants can be described as conscious organisms. The mechanisms involved however are very different.
2. Awareness focuses on behaviour and its degree of complexity rather than arguments about the nervous systems and brains. Awareness provides an operational definition that can provide for measurement.
3. All organisms are aware from the most complex to the most simple. Bacteria are considered conscious (Reber 2018) and intelligent too (Westerhoff et al. 2014). They also exhibit associative learning. This would agree with the IIT assessment (see below). In relative terms, bacterial behaviour is very simple.
4. The diversity of signal sensitivity is also an aspect of awareness. Comparative scales could be drawn up to indicate how different animals and plants are in these respects. It might help make animal scientists more familiar with plant behaviour. A plant organism that can detect the vibrations of a caterpillar jaws chewing, that can determine the species of a pest from the compositions of its salivary juice or that can detect the mating pheromones of a pest and initiating defence responses (Calvo and Trewavas 2020) compares favourably with the familiar animal sensing that leads to running away from a predator.
5. Awareness identifies the similarity and the difference in all life, in terms of selection and environmental modification.

## The integrated information theory (IIT) of consciousness

### Background

Concepts of consciousness are changing, becoming much broader in understanding than the traditional view evoked, for example, by Mallatt et al. (2021) and Taiz et al. (2019). There are at least six major hypotheses of human consciousness which are being competitively matched to try and reduce controversy between the different views (Reardon 2019). The integrated information theory is the first in line for challenge reflecting its significance. As its name implies, the integrated information theory, now a leading theory, emphasises the nature of consciousness solely as information. This view is strongly supported by Earl (2014) who regards everything concerned with consciousness is information.

The significance for plant biologists is that the IIT is blind to any requirement for brains, nerves and synapses as is awareness. The levels of integrated information are on a scale from the most complex in the human brain down to the smallest, a single cell. There are at least four other theories of consciousness that don’t directly require brains or nerves at all (Hameroff 1998; Reber 2018; Friston 2010; Thompson 2007). It is necessary to mention them because Mallatt et al. (2021) omitted to do so.

The IIT was first proposed by Tononi (2004, 2008, 2012). Its origins lie in the research and thinking on human consciousness published from the group around Gerald Edelman and where Tononi was situated. One of Edelman’s primary contributions to understanding primary consciousness was the recognition of loops constructed from re-entrant (recurring) signalling between different regions of the thalamo-cortical region of the human brain, a tissue that interacts mainly with itself (Edelman 2003). And it is the most likely primary anatomical location of human consciousness. Edelman (2003) identifies re-entrant signalling, as the forward-and-back movement of information through functional clusters of neurones.

Tononi and Edelman (1998) raise the issue of consciousness and information in two ways. (1) That the occurrence of one particular conscious state over billions of other possibilities requires a huge amount of information. (2) Functional clusters of neurones can be distinguished by their levels of mutual information. Koch who joined Tononi later on IIT worked for many years with Frances Crick (Koch 2002). These two scientists have created what is currently the most credible theory of consciousness.

### Useful literature

Easily understood versions of the IIT can be found in Koch (2009, 2013, 2014). The most recent fuller versions of the IIT can be found in Oizumi et al. (2014), Tononi (2015) and Tononi et al. (2016). The relationship between fitness and integrated information is described in Edlund et al. (2011) and Joshi et al. (2013). For an extended relatively simple approach, the latter half of Koch,(2019) is recommended. Because the IIT sees consciousness solely as the degree of integrated information, it is blind to synapses, brains and nerves (Tononi and Koch 2015; Koch 2009, 2019).

### The simple basis of IIT

Any network is constructed from the linkages between the constituents. Because the linkages change the behaviour of the constituents, they represent information. The difference between the total information of a system and the information from the isolated system constituents measures its levels of integrated information (Balduzzi and Tononi 2008). It is necessary to distinguish the intrinsic information in an integrated network and Shannon-type, extrinsic information. The latter can be observed and relies on an observer measuring the accuracy with which input signals are transmitted over a noisy channel (Tononi et al. 2016). A procedure for estimating integrated or effective information in subsets of a complex network has been described (Tononi and Sporns (2003); Tononi 2004, 2008).

In a complex, highly integrated network, there is both cause and effect between the various parts, something originally pointed out by the German philosopher Immanuel Kant. The greater the causal power of each part, on the other, the more integrated the network. Highly integrated systems exhibit extensive loss of integrated information with only minor change in the system. The maximal integrated information of any network is defined as ɸ_max_ (Tononi 2008, 2012); humungous in the human brain, but it can be tracked and is thus graded all the way down through the living kingdom to single-celled organisms, the smallest living entities (Tononi and Koch 2015; Koch (2009, 2019) and references above). These single cells also represent the smallest ɸ_max_. If cut in half, they no longer function.

ɸ is also assessed from the present state of the system and how its mechanisms influence the probability of its past and future states (Tononi et al. 2016). The IIT thus explains Barbara McClintock’s 1984 statement that ‘a goal for the future would be to determine the extent of knowledge the cell has of itself and how it uses that knowledge in a thoughtful manner when challenged’. The IIT identifies cell ɸ _max_ as ‘cellular knowledge’.

### Integrated information in plants

The term, *meristem*, is used here to identify the regions of division, tissue specification and then expansion. Similarly the term, *apical meristem*, refers to the dividing zone only. Vertical incisions through the shoot and root meristem create two meristematic halves, neither of which function as meristems. Only with regeneration is function re-established. Consequently two roots and two shoots remain attached to the parent when only one was present before (Calvo et al. 2020a; Cutter 1965; Snow 1942 ). This is precisely the expected behaviour of an area of integrated information. The cambium may join these two meristematic structures since when cut across, the cambial ring is re-established by regeneration from both ends and may exhibit polarity in its regeneration (Snow 1942; Rzimann 1932).

The alterations of apical meristem behaviour(s) are primary sources of change in plant growth and thus behaviour. Meristems receive extrinsic information from other tissues. Together with the current integrated information, a new integrated, intrinsic information nexus is constructed in the apical meristem that represents awareness and leads to molecular changes altering behaviour in turn. Several examples identify the process. Shade avoidance leads to longer, thinner internodes with greater spacing of lateral buds and often altered leaf structure (Casal 2012). Extrinsic information is perceived by the leaves and conveyed to the meristem where the new integrated information nexus modifies genomic expression leading to increased, lateral bud spacing frequency and the rates of cell elongation amongst other changes. Flowering and florigen are another. The dogleg root structure induced by soil stones is another originally reported by Darwin (Massa and Gilroy 2003). Perception here is in the root cap whose combined touch and gravity extrinsic information is conveyed to the apical meristem. Together a new intrinsic information nexus is constructed that codes for a later bend in the root. Competence and coding for later root branching is localised in the meristem (Moreno-Risueno et al. 2010). All these are designed to improve survival via learning and memory; thus, they represent intelligent behaviour too (Karban 2015; Trewavas 2003, 2009; 2014). The cambium acts to assess information from different branches and adjust numbers of vascular elements to competitively increase the distribution of root resources to productive branches and reduce or block root resources to less productive or unproductive branches (Trewavas 2014).

### Potential sources of integrated information in apical meristems

1. The extracellular matrix has the capacity to influence many activities inside cells (Kohorn 2000). Each cell reciprocally interacts with its neighbours and to all others through well-established mechanical connections. Mechanical signalling elevates [Ca^2+^]_i_ (Haley et al. 1995). The contiguous extracellular matrix acts throughout the meristem as intrinsic information. Changes in wall structure change intracellular function (Calvo et al. 2020a).
2. The polarised electrical current that flows through a growing root meristem is known to be essential for maintence of meristem activity (Calvo et al. 2020a). Little is known of the shoot meristem in this regard, but an electrical field is most likely.
3. The complex behaviour surrounding [Ca^2+^]_i_ (Steinhorst and Kudla 2013). Evidence that indicates its location in meristems are the very high levels of calmodulin in the pea root apical meristem. It drops 17 fold on entering expansion with some later recovery (Allan and Trewavas 1985) and it may be attached to the plasma membranes here (Collinge and Trewavas 1989). The first reported plant calcium-activated kinase was located in the pea shoot meristem (Hetherington and Trewavas 1982).

The Ca^2+^ system itself is a very complex network, capable of interpreting complex signal interaction. At least 250 proteins in *Arabidopsis* can bind Ca^2+^, more in other plants. The network is influenced by numerous receptors, including hormones and very large numbers of Ca^2+^-sensitive protein kinases and phosphatases, entry channels and internal Ca^2+^stores that integrate with other aspects of cell control (Steinhorst and Kudla 2013). At least 20 different plant signals use [Ca^2+^]_i_ an electrical signal (Trewavas 2021). Ca^2+^ waves throughout the meristem are one potential global change that changes the present status of integrated information. Initially an external signal acts as extrinsic information. On its initial arrival in the meristem, extrinsic information passes across and through the plasma membrane into cells where it is modified and returns back out in a different form. Each cell modifies what it receives and modifies in turn the extracellular environment interacting with each other. A series of subsequent changes leads to oscillations of [Ca^2+^]_i_ analogous to the recurring signalling in the thalamo-cortical region of the brain constructing integrated information.

There is a relationship between ɸ and the number of different system states, the degree of differentiation, in the meristem (Marshall et al. 2016). These need to be identified. Hoel et al. (2016) raise a further form of information as effective information and the relationship between molecular and integrated conditions.

### Why are assessment mechanisms involved in awareness different between plants and animals?

The difference arises because of the different means of acquiring energy and inevitable competition. Animals almost always have had to move to find food; thus, organs to propel movement evolved. Predator prey relations between animals emphasised the necessary increased speed of movement. Fast nervous systems evolved providing information between sensing and movement through muscles. Eventually a brain evolved to coordinate sensing and movement. Through photosynthesis, plant movement was never essential, but exploiting the local environment required a branching structure and competition for light to drive plant growth upwards. As the plant grows, the potential for more complex behaviour emerges via an increasingly complex vascular system and activation of dormant meristems (Calvo et al. 2017; Mediano et al. 2021). We ourselves are animals and require movement within our time frame to attract attention. That has always led to a downplaying of plant behaviour because it involves slow growth changes that are not immediately apparent. Awareness and assessment are distributed throughout the root and shoot meristems of a growing plant, the only way to efficiently exploit a varying local environment.

## Conclusion

Only mankind possesses real consciousness based on language; for everything else awareness is a suitable better term. Awareness is an operational definition based on behaviour and is present in all life. Using awareness avoids the arguments about plant consciousness arising from nervous systems and brains. Animals and plants use very different mechanisms for awareness and assessment. The extent of awareness varies throughout the living kingdom. It is limited in single cells and has been greatly expanded via multicellularity, the movement to the land and the uncertainty of water availability. Awareness may be simply integrated information. When teaching terms like intelligence and consciousness, simple analogies are useful. There are two kinds of car on present roads. One uses electricity and the other petrol; thus, the mechanisms of transport are entirely different, but movement provides for a similar function going from A to B. Both cars navigate the road, analogous to the function of both animals and plants in navigating the environment, changing behaviour to improve survival. Since plants are the dominant form of life, their mechanisms of awareness are arguably superior to those of animals. There is benefit to plant science to discuss apparent animal terminology. Plant intelligence was posed nearly 20 years ago (Trewavas 2003). Learning, memory and intelligence are now finding its way into natural selection and networks of species developing associative memory (Watson and Szathmary 2016).
